# Exposure to environmentally relevant concentrations of ambient fine particulate matter (PM_2.5_) depletes the ovarian follicle reserve and causes sex-dependent cardiovascular changes in apolipoprotein E null mice

**DOI:** 10.1186/s12989-021-00445-8

**Published:** 2022-01-07

**Authors:** Ulrike Luderer, Jinhwan Lim, Laura Ortiz, Johnny D. Nguyen, Joyce H. Shin, Barrett D. Allen, Lisa S. Liao, Kelli Malott, Veronique Perraud, Lisa M. Wingen, Rebecca J. Arechavala, Bishop Bliss, David A. Herman, Michael T. Kleinman

**Affiliations:** 1 Department of Environmental and Occupational Health, University of California Irvine, 100 Theory Drive, Suite 100, Irvine, CA 92617 USA; 2 Center for Occupational and Environmental Health, University of California Irvine, 100 Theory Drive, Suite 100, Irvine, CA 92617 USA; 3 Department of Developmental and Cell Biology, University of California Irvine, Irvine, CA 92617 USA; 4 Department of Medicine, University of California Irvine, Irvine, CA 92617 USA; 5 Department of Chemistry, University of California Irvine, Irvine, CA 92617 USA

**Keywords:** PM_2.5_, Ovary, Ovarian follicle, Atherosclerosis, Blood pressure, Heart rate variability, Ovariectomy, Sex difference

## Abstract

**Background:**

Fine particulate matter (PM_2.5_) exposure accelerates atherosclerosis and contains known ovotoxic chemicals. However, effects of exposure to PM_2.5_ on the finite ovarian follicle pool have hardly been investigated, nor have interactions between ovarian and cardiovascular effects. We hypothesized that subchronic inhalation exposure to human-relevant concentrations of PM_2.5_ results in destruction of ovarian follicles via apoptosis induction, as well as accelerated recruitment of primordial follicles into the growing pool. Further, we hypothesized that destruction of ovarian follicles enhances the adverse cardiovascular effects of PM_2.5_ in females.

**Results:**

Hyperlipidemic apolipoprotein E (*Apoe*) null ovary-intact or ovariectomized female mice and testis-intact male mice were exposed to concentrated ambient PM_2.5_ or filtered air for 12 weeks, 5 days/week for 4 h/day using a versatile aerosol concentration enrichment system. Primordial, primary, and secondary ovarian follicle numbers were decreased by 45%, 40%, and 17%, respectively, in PM_2.5_-exposed ovary-intact mice compared to controls (*P* < 0.05). The percentage of primary follicles with granulosa cells positive for the mitosis marker Ki67 was increased in the ovaries from PM_2.5_-exposed females versus controls (*P* < 0.05), consistent with increased recruitment of primordial follicles into the growing pool. Exposure to PM_2.5_ increased the percentages of primary and secondary follicles with DNA damage, assessed by γH2AX immunostaining (*P* < 0.05). Exposure to PM_2.5_ increased the percentages of apoptotic antral follicles, determined by TUNEL and activated caspase 3 immunostaining (*P* < 0.05). Removal of the ovaries and PM_2.5_-exposure exacerbated the atherosclerotic effects of hyperlipidemia in females (*P* < 0.05). While there were statistically significant changes in blood pressure and heart rate variability in PM_2.5_-compared to Air-exposed gonad-intact males and females and ovariectomized females, the changes were not consistent between exposure years and assessment methods.

**Conclusions:**

These results demonstrate that subchronic PM_2.5_ exposure depletes the ovarian reserve by increasing recruitment of primordial follicles into the growing pool and increasing apoptosis of growing follicles. Further, PM_2.5_ exposure and removal of the ovaries each increase atherosclerosis progression in *Apoe-/-* females. Premature loss of ovarian function is associated with increased risk of osteoporosis, cardiovascular disease and Alzheimer’s disease in women. Our results thus support possible links between PM_2.5_ exposure and other adverse health outcomes in women.

**Supplementary Information:**

The online version contains supplementary material available at 10.1186/s12989-021-00445-8.

## Background

Particulate matter (PM) air pollution classifications are based on the aerodynamic diameter of the particles, which can be liquid or solid. Fine PM, less than 2.5 μm in diameter (PM_2.5_), are mainly deposited in the lungs. PM_2.5_ varies in composition depending on the sources, geographic location, season and climate. Similar to many other urban areas worldwide, PM_2.5_ from human activities in southern California’s South Coast Air Basin is mainly composed of ammonium nitrate from atmospheric chemical reactions of oxides of nitrogen from mobile (vehicle exhaust) and stationary combustion sources, elemental and organic carbon compounds from combustion sources, and ammonium sulfate from atmospheric chemical reactions of sulfur oxides from combustion sources [1]. PM_2.5_ are regulated in the United States under the Clean Air Act National Ambient Air Quality Standards (NAAQS). However, despite dramatic improvements over the past half century in air quality, the South Coast Air Basin remains in non-attainment of the current PM_2.5_ standard.

The ovarian follicle, which consists of the oocyte surrounded by specialized somatic cells, is the functional unit of the ovary. Ovarian follicles progress from the immature, quiescent primordial follicle stage, through primary, secondary, antral, and preovulatory stages [2]. The somatic granulosa cells form first and are in direct communication with the oocyte. The theca cells differentiate from ovarian stromal cells, forming the outer somatic cell layers of secondary and antral follicles. Unlike the testes, the ovaries do not possess germline stem cells in postnatal life, and thus females are born with a finite complement of oocytes, which are packaged in primordial follicles [3, 4]. Destruction of primordial follicles can therefore cause early onset of ovarian failure. When ovarian failure occurs prior to the age of 40 in women, it is called premature ovarian failure or primary ovarian insufficiency (POF/POI). POF/POI is associated with increased risk of cardiovascular disease, Alzheimer’s disease, and osteoporosis [5–8].

Estradiol synthesis occurs primarily in antral follicles and requires enzymes expressed in the theca cells, which synthesize androstenedione and testosterone, and granulosa cells, which aromatize testosterone to estradiol [9]. Destruction of antral follicles results in decreased estradiol synthesis and disrupted estrous cycles. Apolipoprotein E (*Apoe*) is strongly expressed in ovarian theca cells, interstitial cells, and corpora lutea of rats, mice, and primates [10–14]. APOE regulates androgen synthesis in cultured rat theca cells via regulation of expression of P450 17α-hydroxylase, C17-20 lyase (*Cyp17a1*), the rate-limiting enzyme for androgen synthesis, which converts progesterone to androstenedione [15]. Female *Apoe-/-* mice have been reported to have decreased serum estradiol concentrations compared to non-littermate wild type control females [10].

Epidemiological studies have associated exposure to PM_2.5_ with multiple adverse health effects, including asthma, chronic obstructive pulmonary disease, cardiovascular disease, and adverse pregnancy outcomes [16–18]. Post-menopausal women are at increased risk of cardiovascular disease, and this may be attributed to ovarian dysfunction and reduced ovarian reserve [19–21]. Many experimental studies of atherosclerosis in mice have used the *Apoe-/-* null mouse model, which lacks the APOE cholesterol transport protein that mediates binding of low-density lipoproteins (LDL) and very low-density lipoproteins (VLDL) to LDL receptors in liver and other tissues. Unlike wild type mice, *Apoe-/-* mice develop atherosclerosis [22, 23]. Prior studies showed that exposure to PM_2.5_ accelerates atherosclerosis progression in male *Apoe-/-* mice [24, 25], but this has not been studied in female *Apoe-/-* mice.

The first studies of which we are aware that relate to effects of PM_2.5_ on ovarian function were conducted by one group, who exposed female mice to highly polluted urban air or filtered air for two generations. They found evidence for decreased fertility, increased incidence of irregular estrous cycles, and decreased numbers of antral follicles with exposure to polluted air [26, 27]. Subsequent work by the same group exposed mice during prenatal development and/or postnatally for 1 h daily to diesel exhaust, a major source of PM_2.5_ [28]. They reported that the area of the ovary containing primordial follicles was significantly decreased in mice exposed to diesel exhaust prenatally, postnatally or during both developmental periods compared to filtered air controls. Two recent publications have reported on effects of tracheal instillation of PM_2.5_ [29] or inhalational exposure [30] to PM_2.5_ on ovarian function. Zhou et al. [30] reported an increased percentage of irregular estrous cycles and decreased primordial follicle numbers in mice after 4 months of exposure to ambient PM_2.5_ compared to controls exposed to filtered air. Liao et al. [29] reported increased oxidative DNA damage and apoptosis in the ovaries of mice after tracheal instillation of PM_2.5_. However, no studies have investigated the ovarian effects of exposure to PM_2.5_ in *Apoe-/-* mice despite the importance of APOE in ovarian function.

It is recognized that ambient PM_2.5_ contains polycyclic aromatic hydrocarbons (PAHs) and nitro-PAHs [31]. PAHs are produced during incomplete combustion of organic materials such as fossil fuels, tobacco, wood, and foods. Like PM_2.5_, cigarette smoke also contains dozens of PAHs, and smoking is associated with decreased fertility in women [32, 33]. Consistent with the epidemiological data, exposure of female mice to tobacco smoke caused ovarian follicle loss [34]. Exposure of female mice to PAHs such as benzo[a]pyrene (BaP) or 9,10-dimethyl-1,2-benzanthracene by oral or intraperitoneal routes causes dose-dependent destruction of ovarian primordial, primary, and secondary follicles [35–38]. Inhalation exposure of adult female rats to BaP dose-dependently decreased fertility [39].

Here, we describe rodent inhalation exposure experiments designed to assess the interaction of PM_2.5_ exposure effects on ovarian function and the cardiovascular system. We hypothesized that (1) Subchronic inhalation exposure to human-relevant concentrations of PM_2.5_ results in destruction of ovarian follicles and that this accelerates atherosclerosis in *Apoe-/-* mice. (2) If ovarian follicle destruction enhances atherosclerosis, then removal of the ovaries should enhance atherosclerotic effects of PM_2.5_ to an even greater extent. (3) The mechanism of follicle destruction by PM_2.5_ involves apoptosis of follicles at all stages of development and accelerated recruitment of primordial follicles into the growing pool.

## Results

### Characterization of ambient PM_2.5_

Mice were exposed via whole body exposure to concentrated ambient PM_2.5_ or filtered air (Air) for 5 h per day, 4 days per week for 12 weeks in three cohorts, which were performed in 2017, 2018, and 2019. Particle mass and number concentrations averaged over the 12-week exposures are shown in Table 1 for each year. The average mass concentrations were very similar between the exposure years, 130 ± 5 μg/m^3^ in 2017, 123 ± 11 μg/m^3^ in 2018, and 110 ± 2 μg/m^3^ in 2019. In 2017 gonad-intact male and female mice were studied; in 2018 gonad-intact and ovariectomized (OVAX) female mice were studied; and in 2019 the effect of PM_2.5_ exposure on ovarian estradiol levels in gonad-intact females was studied.

**Table 1:** Particle concentrations averaged over the exposure periods for each year of the study (Means ± standard deviations)

	2017		2018		2019	
	Particle Number (cm^−3^)	Particle Mass (µg m^−3^)	Particle Number (cm^−3^)	Particle Mass (µg m^−3^)	Particle Number (cm^−3^)	Particle Mass (µg m^−3^)
Purified Air	10 ± 20	≤ 5	12 ± 5	≤ 5	83 ± 3	3.5 ± 0.3
Ambient Air	(1.1 ± 0.1) × 10^4^	18 ± 1	(1.5 ± 0.1) × 10^4^	30 ± 8	(1.3 ± 0.1) × 10^4^	27 ± 0.2
PM_2.5_	(8.6 ± 2.0) × 10^4^	130 ± 5	(9.5 ± 2.0) × 10^4^	123 ± 11	(8.3 ± 0.5) × 10^4^	110 ± 2

We estimated the total exposure to PAHs in PM_2.5_ during the course of the 12 week exposure by first calculating the total amount of air inhaled and exhaled: minute ventilation of 1.46 ml per gram body weight [40] × 20 g body weight × 300 min per 5 h exposure session × 48 sessions × 10^–6^ m^3^/ml = 0.42 m^3^. A representative estimation of PAHs in particles to which mice in this study were exposed was based on previous studies performed close to heavily trafficked roadways in Los Angeles, in which about 2 ng/m^3^ of low molecular weight PAHs and 2 ng/m^3^ of high molecular weight PAHs were measured in concentrated ambient PM_2.5_ [41, 42]. Therefore, we estimated that the mice could have been exposed to about 1.7 ng of total PAHs over the course of the entire 12-week exposure. This translates to 85 ng/kg for a 20 g mouse.

### Exposure to PM_2.5_ disrupted estrous cycling

There were no statistically significant differences in estrous cycling or in ovarian follicle counts in the Air exposed control mice during the 2017 and 2018 exposure years, so the estrous cycle and follicle count endpoints from both years were combined for analyses. Effects of 12-week exposure to concentrated ambient PM_2.5_ on estrous cycling are summarized in Table 2. Mice exposed to PM_2.5_ were statistically significantly more likely to have irregular estrous cycles, defined as any cycles shorter than 4 days or longer than 5 days, than control mice (*P* = 0.008, Fisher’s exact test). 35% of PM_2.5_-exposed mice had irregular cycles, while all the control mice had regular 4- to 5-day cycles. Estrous cycles were slightly, but non-statistically significantly longer in mice exposed to PM_2.5_. The percentage of days with leukocytic vaginal cytology, characteristic of metestrus and diestrus, was statistically significantly decreased in mice exposed to PM_2.5_ (*P* = 0.027, 2-way ANOVA), while the percentage of days with predominantly cornified cytology, characteristic of estrus, was non-significantly increased.

**Table 2:** Effects of exposure to PM_2.5_ on estrous cycles

	Filtered air^a^	PM_2.5_^a^
Cycle length (days)	4.3 ± 0.1	4.6 ± 0.1
% with abnormal cycles^b^	0	35
% days with leukocytic cytology^c^	39.2 ± 1.4	33.0 ± 2.3
% days with cornified cytology	28.1 ± 1.3	32.8 ± 3.4

^a^N = 20/group

^b^Not having 4 or 5 day cycles, *P* = 0.008, Fisher’s exact test

^c^*P* = 0.027, 2-way ANOVA

### Exposure to PM_2.5_ decreased ovarian follicle numbers

**Fig. 1:**
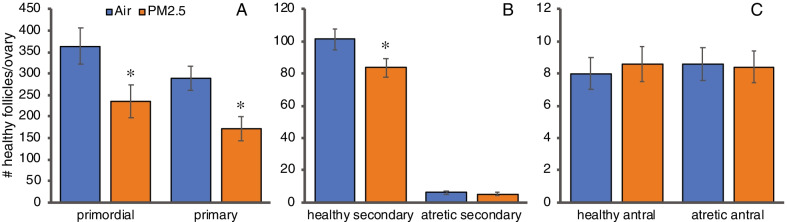
Effects of PM_2.5_ exposure on ovarian follicle numbers: 3-month old female mice were exposed to concentrated ambient PM_2.5_ or filtered air 4 h per day, 5 days per week for 12 weeks and were euthanized 24 h after the last exposure day for enumeration of ovarian follicles as described in Methods. Graphs show the means ± SEM number of follicles per ovary. **A** Healthy primordial and primary follicle numbers were significantly decreased in PM_2.5_ exposed mice compared to air controls. **B** Healthy, but not atretic, secondary follicle numbers were significantly decreased in PM_2.5_ exposed mice compared to air controls. **C** Neither healthy, nor atretic antral follicle numbers were significantly changed in PM_2.5_ exposed mice compared to air controls. *P < 0.05 compared to air controls. N = 20/group

Exposure to ambient PM_2.5_ decreased the numbers of ovarian primordial follicles by 45% (*P* = 0.031, 2-way ANOVA; Fig. 1A) and primary follicles by 40% (*P* = 0.006, 2-way ANOVA; Fig. 1A). Healthy secondary follicle numbers were significantly decreased by 17% (*P* = 0.048, 2-way ANOVA; Fig. 1B), while healthy antral follicles and atretic secondary and antral follicle numbers were not affected (Fig. 1B). Corpora lutea numbers also were not affected (data not shown).

### Exposure to PM_2.5_ increased DNA damage in ovarian follicles

**Fig. 2:**
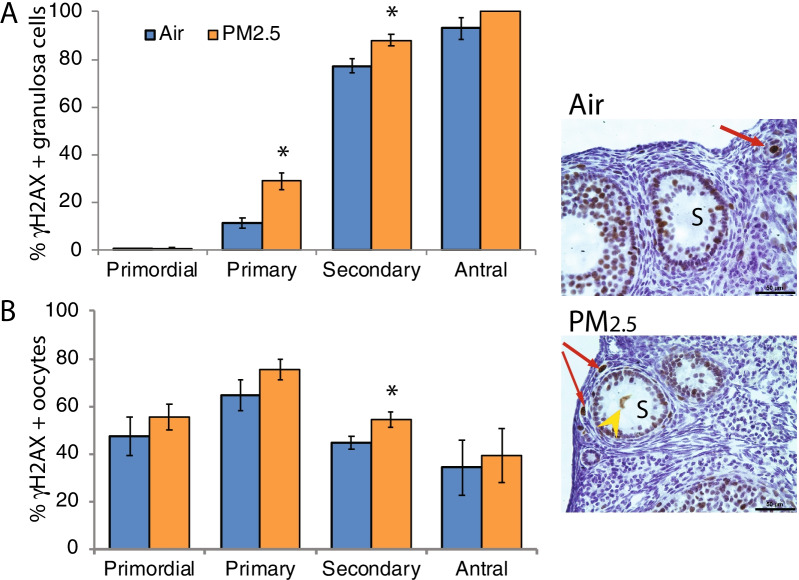
Effects of PM_2.5_ exposure on DNA damage in ovarian follicles: Female mice were exposed to concentrated ambient PM_2.5_ or filtered air as described for Fig. 1 and ovaries were processed for immunostaining for γH2AX as described in Methods. The graphs show the means ± SEM percentages of follicles with γH2AX-positive granulosa cells (**A**) or oocytes (**B**). Representative photomicrographs taken with 40 × objective show secondary follicles (S) with γH2AX-positive, brown-stained nuclei of granulosa cells and oocyte (yellow arrow; the nucleus does not appear round because of fixation artifact) and primordial and primary follicles with γH2AX-positive oocytes (red arrows). PM_2.5_ exposure significantly increased the percentages of primary and secondary follicles with γH2AX-positive granulosa cells (**A**) and of secondary follicles with γH2AX-positive oocytes (**B**). *P < 0.05 compared to air controls. N = 6–7/group. Scale bars, 50 μm

We performed immunostaining for γH2AX, which mediates recruitment of DNA repair proteins to sites of DNA damage and is a marker for DNA damage [43–45], on ovaries from the 2017 cohort. There was a statistically significant increase in the fraction of primary follicles with γH2AX-positive granulosa cells (*P* = 0.004, Mann Whitney test; *P* = 0.001, t-test) and in the fraction of secondary follicles with γH2AX-positive granulosa cells (*P* = 0.022, Mann Whitney test; *P* = 0.017, t-test), while the fractions of primordial and antral follicles with γH2AX-positive granulosa cells were unchanged by PM_2.5_ exposure (Fig. 2A). There was a statistically significant increase in the fraction of secondary follicles with γH2AX-positive oocytes in PM_2.5_ exposed mice (*P* = 0.040, t-test; *P* = 0.086, Mann Whitney test), and no effect of PM_2.5_ exposure on percentages of primordial, primary, or antral follicles with γH2AX-positive oocytes (Fig. 2B).

### Exposure to PM_2.5_ increased the percentage of apoptotic antral follicles

**Fig. 3:**
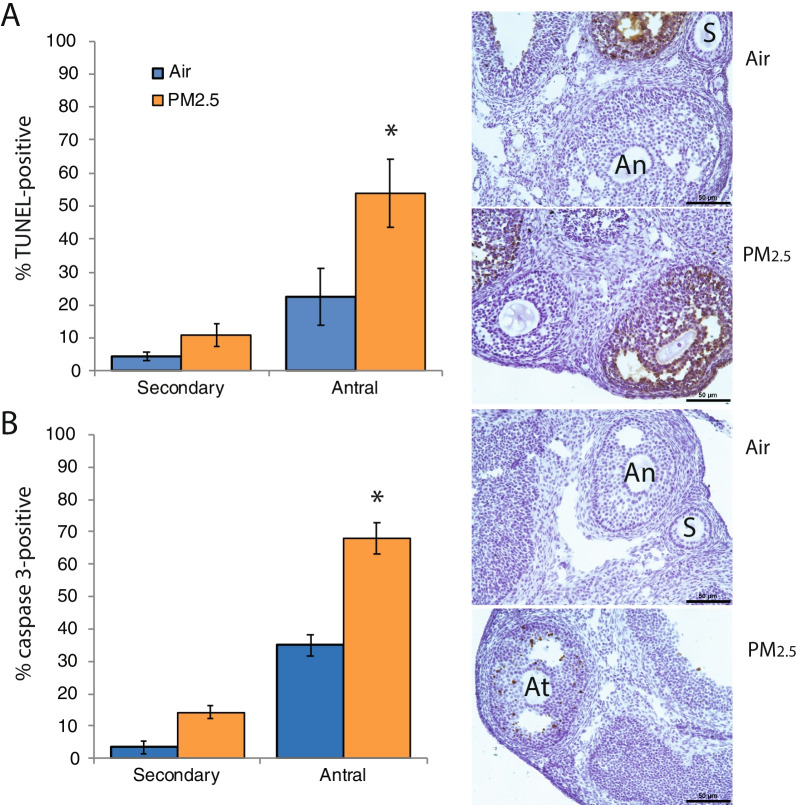
Effects of PM_2.5_ exposure on apoptosis in ovarian follicles. Female mice were exposed to concentrated ambient PM_2.5_ or filtered air as described for Fig. 1 and ovaries were processed for TUNEL or immunostaining for activated caspase 3 as detailed in Methods. The graphs show the means ± SEM percentage of TUNEL- (**A**) or activated caspase 3- **B** positive secondary and antral follicles. **A** Representative photomicrographs taken with 20 × objective show TUNEL-positive antral follicles with brown stained nuclei in granulosa cells, as well as TUNEL-negative secondary (S) and antral (An) follicles in air control and PM_2.5_ ovaries. PM_2.5_ exposure significantly increased the percentage of antral follicles with TUNEL-positive granulosa cells. **B** Representative photomicrographs taken with 20 × objective show activated caspase 3-positive antral follicles with brown stained nuclei in granulosa cells (At), as well as caspase 3-negative secondary (S) and antral (An) follicles in air control ovaries. PM_2.5_ exposure significantly increased the percentage of antral follicles with activated caspase 3-positive granulosa cells. *P < 0.05 compared to air controls. N = 6–7/group. Scale bars, 50 μm

TUNEL was used to identify follicles in late stages of atresia (apoptotic degeneration) in ovaries from the 2017 cohort. Ovaries from PM_2.5_ exposed mice had statistically significantly increased percentages of antral follicles with TUNEL-positive granulosa cells (*P* = 0.036 Mann Whitney test; *P* = 0.058, t-test; Fig. 3A) and percentages of antral follicles with activated caspase 3- positive granulosa cells (*P* = 0.032 Mann Whitney test; *P* = 0.065, t-test; Fig. 3B). There were no statistically significant exposure-related differences in the percentages of TUNEL-positive secondary follicles or of activated caspase 3-positive secondary follicles (Fig. 3A, B). TUNEL- or activated caspase 3- positive primordial and primary follicles were very rarely observed in either experimental group (data not shown).

### Exposure to PM_2.5_ increased primordial follicle recruitment

**Fig. 4:**
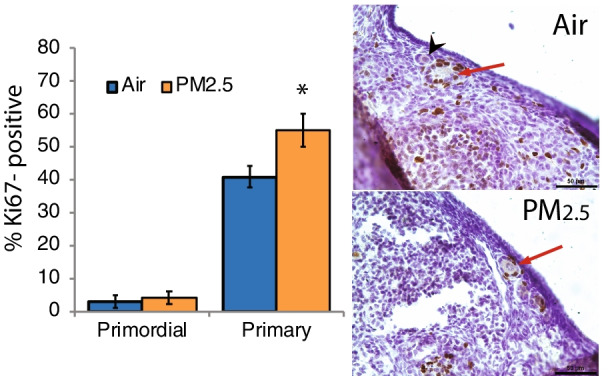
Effects of PM_2.5_ exposure on activation of primordial ovarian follicles. Female mice were exposed to concentrated ambient PM_2.5_ or filtered air as described for Fig. 1 and ovaries were processed for immunostaining for the proliferation protein Ki67 as detailed in Methods. Representative photomicrographs taken with 40 × objective show Ki67-positive follicles with brown stained granulosa cell nuclei in air control and PM_2.5_ ovaries. Red arrows point to primary follicles with Ki67-positive granulosa cells. Black arrowhead points to primordial follicle with Ki67-negative granulosa cells. Scale bars, 50 μm. The graph shows the means ± SEM percentages of Ki67-positive primordial and primary follicles. PM_2.5_ exposure significantly increased the percentage of primary follicles with Ki67-positive granulosa cells. *P < 0.05 compared to air controls. N = 6/group

We utilized immunostaining for Ki67 to identify actively dividing granulosa cells in follicles in ovaries from the 2017 cohort. Ki67 protein is expressed throughout the cell cycle, but not during G_0_ [46]. Compared to filtered air exposed mice, ovaries from PM_2.5_-exposed mice had statistically significantly increased percentage of primary follicles with Ki67-positive, mitotic granulosa cells, consistent with increased recruitment of primordial follicles into the growing pool (*P* = 0.040, t-test; *P* = 0.055, Mann Whitney test; Fig. 4). Essentially all secondary and antral follicles had numerous Ki67-positive granulosa cells regardless of PM_2.5_ exposure, as expected in these rapidly growing follicles (Fig. 4 representative images).

### Exposure to PM_2.5_ did not affect ovarian content of estradiol

Estradiol concentrations were measured in extracts of whole, proestrous ovaries from the 2018 and 2019 cohorts, which were derivatized with 1,2-dimethylimidazole-5-sulfonyl chloride (DMIS) and quantified using LC–MS/MS. The difference in ovarian estradiol content between ovaries of mice exposed to Air and PM_2.5_ was not statistically significant (Table 3). Ovarian estradiol content also did not differ by exposure year (2018 or 2019) or whether the animal had an implant. Some mice had cycles of variable lengths; in those cases, one could not be sure that the mouse was euthanized on proestrus, despite vaginal cytology consistent with proestrus. There was a statistically significant difference in ovarian estradiol content between mice with all cycles of the same length compared to mice with variable length cycles by t-test and in a 2-way ANOVA with exposure, with higher estradiol content in mice with variable length cycles. The effect of exposure was not statistically significant in either analysis. Interestingly, the estradiol contents of ovarian extracts from unexposed wild type mice euthanized on proestrus (the cycle stage with highest estradiol concentrations), were significantly higher than the estradiol content of ovaries from either control or PM_2.5_ exposed *Apoe-/-* mice (*P* = 0.038, Mann Whitney test; *P* = 0.049, t-test, wild type compared to Air exposed *Apoe-/-* mice; *P* = 0.021, Mann Whitney; *P* = 0.010 t-test, wild type compared to PM_2.5_ exposed *Apoe-/-* mice). The latter results suggest an effect of *Apoe* deletion on ovarian estradiol synthesis.

**Table 3:** Effects of exposure to PM_2.5_ on ovarian estradiol content

	*Apoe-/-* Air	*Apoe-/-* PM_2.5_	Wild type unexposed
N	26	24	3
Estradiol (pg/mg ovary)	20.7 ± 12.0	8.8 ± 3.9	38.7 ± 21.9

### Plaque formation in arteries of SHAM and OVAX mice

**Fig. 5:**
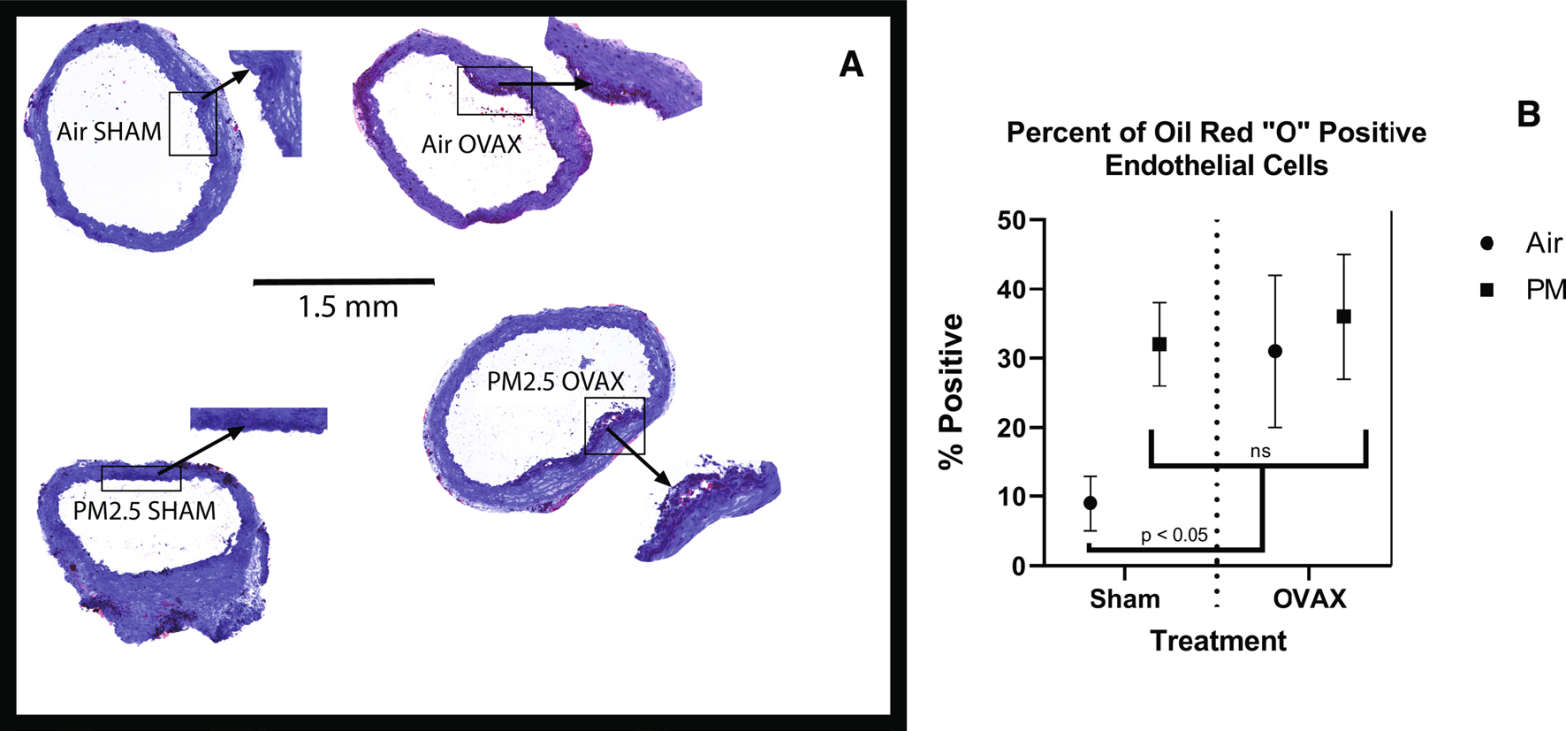
Effects of ovariectomy and PM_2.5_-exposure on atherosclerosis. **A** Representative images of atherosclerotic plaques in Air- and PM_2.5_-exposed and ovariectomized (OVAX) and ovary intact (Sham) mice. Cross-sections of aortic root were stained with the lipid stain Oil Red O and counterstained with hematoxylin. Scale bar 1.5 mm for all four aortic root cross-sections. Areas within rectangles are magnified in insets. **B** Means ± SEM percentages of Oil Red O-positive endothelial cell area. ns, no significant difference among Sham PM_2.5_, OVAX Air, and OVAX PM_2.5_; all three groups differed significantly (*P* < 0.05) from Sham Air. N = 3–4/group

Aortic arch cross-sections from ovariectomized (OVAX) mice or mice that underwent a sham surgery prior to being exposed to Air or PM_2.5_ for 12 weeks were stained with Oil Red O and counterstained with hematoxylin. Typical sections are shown in Fig. 5A. Air and PM_2.5_-exposed OVAX mice and PM_2.5_-exposed SHAM mice had greater atherosclerosis progression than ovary-intact SHAM controls, as indicated by the percentage of endothelial cell area staining positive with Oil Red ‘O’ dye (Fig. 5B). We note that the effects of both interventions were not additive; plaque area increases in PM_2.5_-exposed OVAX mice were slightly higher than in PM_2.5_ SHAM mice, but the difference was not statistically significant. PM_2.5_ exposure reduced the percent area taken up by the lumen in SHAM mice compared to Air exposed SHAM; this difference approached statistical significance (Table 4; *P* = 0.051).

**Table 4:** Effects of exposure to PM_2.5_ and ovariectomy on aortic arch lumen area

Area	Air OVAX	PM_2.5_ OVAX	Air SHAM	PM_2.5_ SHAM
Lumen	3.84 ± 0.47	3.00 ± 0.31	3.21 ± 0.29	2.96 ± 0.28
Wall + Plaque + Lumen	6.54 ± 0.50	5.68 ± 0.43	5.30 ± 0.38	5.96 ± 0.11
% lumen area	0.58 ± 0.03	0.59 ± 0.02	0.60 ± 0.02	0.50 ± 0.04*

N = 3–4/group

*P = 0.051, compared to Air SHAM

### Blood pressure

Baseline blood pressures were measured for one week, the week prior to actual PM exposures while the mice were breathing purified air. Baseline measurements might be expected to be somewhat elevated since the mice, although acclimated to the system for one week prior to the baseline, might still have been not completely used to the level of handling and confinement. Blood pressure data were acquired during the final 6 weeks of the exposure (weeks 6–11), daily averaged over 4 h via telemetry in mice implanted with transmitters, and via tail cuff once per week in mice with and without implanted transmitters in 2017 and in mice without transmitters only in 2018. Blood pressure data are presented in Table 5 and the changes in blood pressure during the final weeks of exposure compared to baseline are presented in Additional file 1: Table S1.

**Table 5:** Intra-arterial and tail cuff systolic blood pressure (SBP) and diastolic blood pressure (DBP) in male and female *Apoe-/-* mice, 2017 cohort^#^

		Male			Female		
		Air	PM_2.5_	*P-value	Air	PM_2.5_	*P-value
		Transmitter—Intra-arterial					
Baseline	SBP	116.4 ± 1.0	119.1 ± 0.8	0.340	114.4 ± 2.8	117.3 ± 0.5	0.101
	DBP	96.7 ± 1.2	101.6 ± 1.0	0.003	97.6 ± 2.5	98.8 ± 0.7	0.271
Weeks 6–11	SBP	114.0 ± 1.4	117.2 ± 0.7	0.033	101.2 ± 1.7	122.7 ± 0.7	< 0.001
	DBP	95.9 ± 0.9	97.6 ± 0.5	0.089	84.3 ± 1.6	102.6 ± 0.9	< 0.001
		Transmitter—Tail Cuff					
Baseline	SBP	123.8 ± 5.7	139.4 ± 9.8	0.203	143.7 ± 4.6	123.4 ± 8.5	0.070
	DBP	96.5 ± 2.7	111.2 ± 7.8	0.115	112.7 ± 3.0	96.6 ± 8.3	0.105
Weeks 6–11	SBP	129.0 ± 4.1	135.7 ± 4.3	0.264	145.0 ± 5.7	132.3 ± 8.5	0.099
	DBP	98.8 ± 4.1	105.7 ± 4.3	0.248	114.3 ± 5.0	103.8 ± 8.3	0.133
		Non-transmitter—Tail Cuff					
Baseline	SBP	145.4 ± 4.9	139.3 ± 6.0	0.445	127.9 ± 4.2	135.5 ± 4.4	0.226
	DBP	116.6 ± 4.3	103.2 ± 4.2	0.038	100.5 ± 4.4	105.5 ± 4.0	0.404
Weeks 6–11	SBP	140.1 ± 6.5	139.8 ± 6.2	0.942	139.2 ± 9.3	140.1 ± 4.4	0.817
	DBP	109.3 ± 5.4	109.1 ± 5.2	0.950	112.2 ± 7.5	111.2 ± 4.0	0.769

N = 10/experimental group, Non-transmitter; N = 4–5/experimental group, Transmitter

*P-values are for PM_2.5_ versus Air of same sex and BP measurement method, t-test

^***#***^Blood pressures are in mmHg

At baseline prior to PM_2.5_ exposure, the 2017 cohort transmitter males to be exposed to PM_2.5_ had a higher diastolic blood pressure (DBP) than males allocated to the Air group, while there was no significant difference at baseline in diastolic blood pressure for transmitter females or in systolic blood pressure for either sex (Table 5). Despite systolic blood pressure (SBP) dropping in all transmitter males from baseline measures, PM_2.5_ males had significantly higher SBP, and a trend of higher DBP compared to Air males as well (Table 5). The decrease in DBP from baseline, but not the decrease in SBP, differed significantly between Air and PM_2.5_-exposed males (Additional file 1: Table S1). Air-exposed females exhibited a drop in SBP and DBP during weeks 6–11 compared to baseline, while SBP and DBP increased from baseline in PM_2.5_-exposed females (Table 5 and Additional file 1: Table S1). Both SBP and DBP during the final 6 weeks of exposure were significantly increased in PM_2.5_ exposed females compared to air controls (Table 5), and the changes in SBP and DBP from baseline also differed significantly between Air and PM_2.5_-exposed females (Additional file 1: Table S1). The effects of PM_2.5_ exposure were broadly consistent in both males and females when comparing intra-arterial and tail cuff blood pressures from transmitter-implanted mice (top two sections of Table 5). Table 5 also shows that effects of PM_2.5_ exposure differed based on blood pressures obtained via tail cuff from the transmitter mice compared to the non-implanted mice. In contrast to the increased blood pressure observed in transmitter females, there were no statistically significant differences in SBP or DBP between PM_2.5_ and Air-exposed non-implanted females during the final 6 weeks of exposure.

For the 2018 cohort, there were again differences in the effects of PM_2.5_ depending on whether the mice had telemetry implants or not. In mice with implants, there was no effect of chronic PM_2.5_ exposure on SBP or DBP (Table 6) or on the change in SBP or DBP from baseline (Additional file 1: Table S2) in ovariectomized female ApoE-/- mice. In the SHAM group at baseline the mice to be exposed to PM_2.5_ had significantly higher SBP. However, over the course of the exposure SBP and DBP dropped in PM_2.5_ animals and rose in the Air group. This led to a significant difference in both systolic and diastolic blood pressure between PM_2.5_- and air-exposed SHAM animals, with PM_2.5_-exposure leading to a decrease in SBP and DBP compared to Air-exposure and a decrease in SBP and DBP from baseline (Additional file 1: Table S2) in the ovary-intact SHAM animals. In contrast, in the non-implanted mice in which blood pressure was measured by tail cuff, there were no statistically significant differences in SBP or DBP between PM_2.5_- and Air-exposed SHAM mice, while SBP and DBP both increased in ovariectomized mice exposed to PM_2.5_ compared to Air. The lack of a statistically significant effect of PM_2.5_ exposure on either SBP or DBP measured by tail cuff was consistent for ovary-intact, non-implanted females in both the 2017 and 2018 cohorts (Tables 5 and 6). Ovariectomy significantly decreased the change in SBP and increased the change in DBP from baseline in Air-exposed mice, while it significantly increased the changes in SBP and DBP from baseline in PM_2.5_-exposed mice (*P* < 0.001; Additional file 1: Table S3).

**Table 6:** Intra-arterial and tail cuff systolic blood pressure (SBP) and diastolic blood pressure (DBP) in OVAX and SHAM female ApoE-/- mice, 2018 cohort

			OVAX			SHAM		
			Air	PM_2.5_	*P*-value	Air	PM_2.5_	*P*-value
			Transmitter—Intra-arterial					
Baseline	SBP	mmHg	122.8 ± 1.3	121.3 ± 1.5	0.454	123.5 ± 0.9	127.4 ± 1.3	0.020
	DBP	mmHg	99.3 ± 2.2	95.6 ± 1.4	0.139	99.2 ± 1.2	99.6 ± 1.0	0.778
Weeks 6–11	SBP	mmHg	123.6 ± 0.6	123.7 ± 0.6	0.931	129.8 ± 0.5	122.4 ± 1.1	< 0.001
	DBP	mmHg	102.0 ± 1.0	100.7 ± 0.6	0.242	101.3 ± 0.5	96.6 ± 0.9	< 0.001
			Non-transmitter—Tail Cuff					
Baseline	SBP	mmHg	125.2 ± 3.8	131.7 ± 4.8	0.302	136.9 ± 5.4	133.6 ± 3.2	0.609
	DBP	mmHg	103.1 ± 3.1	107.9 ± 2.9	0.274	109.3 ± 4.5	108.9 ± 3.0	0.946
Weeks 6–11	SBP	mmHg	134.8 ± 2.8	145.3 ± 3.4	0.018	140.4 ± 2.4	138.5 ± 2.8	0.602
	DBP	mmHg	111.1 ± 2.5	119.1 ± 3.2	0.054	113.2 ± 2.4	112.5 ± 2.4	0.851

*P*-values are for PM_2.5_ versus Air of same group and BP measurement method, t-test

N = 10, Non-transmitter; N = 4–5, Transmitter

Blood pressure measurements obtained via tail cuff were consistently higher than intra-arterial blood pressures obtained via telemetry, whether obtained from mice with or without implants (Tables 5 and 6). The differences in SBP obtained via telemetry versus tail cuff from the same mice were statistically significant for Air-exposed males and females (*P* = 0.015, *P* < 0.001, respectively) at baseline and weeks 6–11 (*P* = 0.002, *P* < 0.001, respectively), as well as for PM_2.5_-exposed males during weeks 6–11 (*P* < 0.001; Table 5). The differences in DBP obtained via telemetry versus tail cuff from the same mice were statistically significant for Air-exposed females at both baseline and weeks 6–11 (*P* = 0.017, *P* < 0.001, respectively; Table 5). There were also statistically significant differences in tail cuff blood pressures between mice within the same exposure group depending on whether they had telemetry implants or not. Both Air-exposed males and females had significantly different SBP at baseline by transmitter status, and Air exposed males also had significantly different DBP at baseline by transmitter status (Table 5). Interestingly, the tail cuff SBP and DBP at baseline were lower in the males with transmitters, while in the females they were higher in those with transmitters. By the second half of the exposure period, there were no longer any tail cuff blood pressure differences in females by transmitter status. In males, the lower SBP and DBP in mice with transmitters compared to those without approached significance in the second half of the exposure period (*P* = 0.057 and *P* = 0.065, respectively).

### Heart rate variability

During the 2017 exposures, heart rate (HR) did not change significantly in response to PM_2.5_ in either male or female mice (Table 7). Over the course of the exposure, there was a general tendency towards decreased HR variability (HRV) in all PM_2.5_ exposed mice relative to their Air controls, but there were differences between males and females. SDNN did not show a significant difference in males during the last 6 weeks of the exposure, but the decrease in SDNN was clear in PM_2.5_-exposed females. Significant decreases in RMSSD in both males and females reflect changes to parasympathetic nervous system inputs to the heart after sub-chronic PM_2.5_ exposure. Sex-differences are evident in the PM_2.5_-induced HRV changes, with females showing a greater negative HRV response than males.

**Table 7:** Percent change from baseline ± SEM in heart rate variability measures during weeks 6–11 in male and female ApoE-/- mice, 2017 cohort

		Male			Female		
		Air	PM_2.5_	*p*-value	Air	PM_2.5_	*P*-value
HR	*% change*	2.3 ± 0.7	1.6 ± 0.5	0.396	7.1 ± 0.7	7.5 ± 1.3	0.786
SDNN	*% change*	3.2 ± 2.4	1.5 ± 1.4	0.527	-2.8 ± 2.2	-12.5 ± 2.3	0.002
RMSSD	*% change*	12.7 ± 3.8	-3.6 ± 2.5	< 0.001	17.4 ± 8.1	-12.5 ± 2.1	0.001

*P*-values are for PM_2.5_ versus Air of same sex and BP measurement method, t-test

HR, heart rate; SDNN, standard deviation of normal RR intervals; RMSSD, root mean squared of successive differences of normal RR intervals

PM_2.5_ exposure in the 2018 cohort led to significantly higher HR in both OVAX and SHAM groups (Table 8). During the final six weeks of the study, SDNN was more negative in the ovariectomized animals, but was not significantly affected by exposure in either group. RMSSD was significantly higher in PM_2.5_-exposed OVAX animals and trended similarly in the SHAM PM_2.5_ group, although not reaching significance. The increase in RMSSD in PM_2.5_-exposed mice indicates an increase in parasympathetic response.

**Table 8:** Percent change from baseline ± SEM in heart rate variability measures during weeks 6–11 in OVAX and SHAM female ApoE-/- mice, 2018 cohort

		OVAX			SHAM		
		Air	PM_2.5_	*P*-value	Air	PM_2.5_	*P*-value
HR	*% change*	2.6 ± 0.5	3.6 ± 0.5	0.046	2.8 ± 0.5	4.9 ± 0.5	< 0.001
SDNN	*% change*	− 4.6 ± 1.3	− 5.0 ± 1.2	0.142	2.8 ± 1.4	− 1.1 ± 1.4	0.134
RMSSD	*% change*	− 2.9 ± 1.5	2.0 ± 1.6	0.027	− 8.3 ± 2.7	− 0.9 ± 1.7	0.285

*P*-values are for PM_2.5_ versus Air of same group, t-test

HR, heart rate; SDNN, standard deviation of normal RR intervals; RMSSD, root mean squared of successive differences of normal RR intervals

## Discussion

Our data show for the first time that inhalational exposure to concentrated ambient PM_2.5_ at concentrations to which humans are exposed depletes the ovarian follicle reserve in female *Apoe-/-* mice. Our data show that exposure for 5 h per day, 4 days per week for 12 weeks to average concentrations of 120–130 µg/m^3^ decreased the irreplaceable reserve of ovarian primordial and primary follicles by 45% and 40%, respectively. More mature secondary follicles were decreased by 17%. Our data further support that these decreases in follicle numbers are caused by accelerated recruitment of primordial follicles into the growing pool and DNA damage-induced apoptosis of growing follicles. PM_2.5_-exposed female mice were also significantly less likely to have normal estrous cycles, defined as 4 to 5 days in length. Atherosclerotic plaques developed in all mice as expected, and PM_2.5_-exposed ovary-intact and ovariectomized mice and filtered air-exposed ovariectomized mice had larger plaque area than filtered air-exposed controls. Blood pressure and heart rate variability were significantly affected by PM_2.5_ exposure in gonad-intact males and females, as well as in ovariectomized female mice.

The 24-h time-weighted average PM_2.5_ concentrations to which the mice in this study were exposed averaged 27 to 29 μg/m^3^, below the US NAAQS 24-h average value for PM_2.5_ of 35 μg/m^3^ (98th percentile averaged over three years; [47]). Peak PM_2.5_ concentrations, above 130 μg/m^3^ for 2 to 4 h are often observed over wide areas during wildfire episodes, such as the large fires in California and Oregon during September 2020 (https://gispub.epa.gov/airnow; [48]). Moreover, 24-h time-weighted average concentrations of 130 μg/m^3^ or higher are common in cities in India, China, Pakistan, Bangladesh, and other nations [49, 50]. Importantly, size analyses of the exposure atmospheres in our study made using an SMPS (TSI, Inc.) demonstrated that the mobility diameters of the vast majority of particles in the concentrated ambient PM_2.5_ were less than 160 nm, and most of the particles were in the ultrafine range (< 100 nm; data not shown). Ultrafine particles are mainly deposited in the alveoli [51]. However, because of their small size, they are not efficiently cleared from the alveoli by macrophages and are able to penetrate across the lung interstitium and translocate into the circulation to reach other target organs [51, 52]. In addition, the ultrafine fraction of PM is enriched in organic substances, such as PAHs [24].

Most physiological ovarian follicle exhaustion in the adult ovary occurs after recruitment of primordial follicles into the growing pool. Once recruited, a follicle can develop to the preovulatory stage or die along the way. Only follicles that have reached the preovulatory stage at the time of the preovulatory surge of gonadotropins are capable of ovulating in response to the surge. More than 95% of follicles do not reach the preovulatory stage and undergo a process of degeneration called atresia. This occurs predominantly at the antral stage, when follicles are dependent on gonadotropin support for survival [53–56]. Atresia of antral follicles is characterized by apoptotic death of the granulosa cells, which ultimately also results in death of the oocyte. Apoptotic death of antral follicles can be induced via activation of the intrinsic (mitochondrial) or extrinsic (receptor-mediated) pathways, both of which converge on activation of caspase 3 [56]. Exposure to toxicants that induce apoptosis at any stage of follicle development and/or that accelerate recruitment of primordial follicles into the growing pool can accelerate the exhaustion of the ovarian follicle pool, resulting in premature ovarian failure [57]. Ionizing radiation and anticancer drugs are among the most investigated ovarian toxicants. Many of these agents initiate apoptosis in ovarian follicles by directly or indirectly (via generation of reactive oxygen species, ROS) damaging DNA [57]. Our prior work showed that the mutagenic PAH 9,10-dimethyl-1,2-benzanthracene increases ROS generation and protein levels of the proapoptotic BCL-2 family member BAX and activated caspase 3 in granulosa cells of cultured rat antral follicles [58]. These prior findings suggested to us that exposure to PM_2.5_, as a source of PAHs, may cause follicle depletion via similar mechanisms. The present results show that exposure to PM_2.5_ increased the percentage of primary follicles that are positive for the mitosis marker Ki67. Because we grouped transitional follicles (those that display characteristics of both primary and primordial follicles) with primary follicles when enumerating follicles, these data provide evidence for increased recruitment of primordial follicles into the growing pool. Accelerated recruitment of primordial follicles could be related to the decreased number of secondary follicles. Prior work has shown that depletion of secondary follicles by treatment with the anticancer drug cyclophosphamide results in decreased ovarian mRNA expression of Anti-Müllerian Hormone (AMH) [59, 60]. AMH produced by growing follicles normally inhibits primordial follicle recruitment, and lower AMH levels result in accelerated recruitment of primordial follicles [61, 62]. The present results also demonstrate that exposure to PM_2.5_ increased DNA damage, measured by γH2AX immunostaining in primary and secondary follicles, and increased apoptosis, measured by activated caspase 3 immunostaining and TUNEL, in antral follicles. PM_2.5_ contains numerous PAHs, metabolic activation of which leads to formation of bulky DNA adducts that result in phosphorylation of H2AX [63]. For example, benzo[a]pyrene diol epoxide, *o-*quinone, and radical cation metabolites react with DNA to form bulky adducts, and the *o*-semiquinone and *o*-quinone metabolites can undergo redox cycling, leading to formation of reactive oxygen species and oxidative DNA damage [64]. Chromium and arsenic are carcinogenic metals found in PM_2.5_, both of which cause oxidative stress and induce oxidative DNA damage [65, 66]. Arsenic has also been shown to disrupt multiple DNA repair pathways [66]. We did not observe evidence of apoptosis in primordial or primary follicles; however, it is possible that other cell death mechanisms may be at play in these follicles. For example, a recent study showed that exposure to diesel exhaust particle extract decreased autophagic turnover and decreased neuron numbers in the brains of zebrafish and that stimulation of autophagic turnover rescued the decrease in neurons [67].

We estimated that the mice in our study were exposed to 85 ng/kg body weight of total PAHs during the exposure period, based on representative PAH content for ambient particles in the southern California air basin. Compared to prior experimental work with individual PAHs in this and other laboratories, this is orders of magnitude lower than the ED_50_ for primordial follicle depletion (dose at which 50% of primordial follicles are depleted) by individual PAHs administered via intraperitoneal injection [37, 38, 68]. For example, the ED_50_ values for primordial follicle depletion for three different PAHs (benzo[a]pyrene, 7,12-dimethylbenz[a]anthracene, and 3-methylcholanthrene) in C57BL/6 mice dosed daily for 15 days by intraperitoneal injection were two to four orders of magnitude higher than the estimated dose of total PAHs in the current study [37]. For a single intraperitoneal injection of the same PAHs, the ED_50_ for primordial depletion was four to five orders of magnitude higher than the estimated dose of total PAHs in the current study [68]. Acknowledging the different route of administration in these prior studies, these comparisons nonetheless lead to the conclusion that the ovotoxicity of PM_2.5_ observed in our study is likely not due solely to its PAH content. PM_2.5_ is a complex mixture, which contains other known ovotoxicants, such as the metals arsenic and chromium [42, 69–71], as well as many compounds that have not been investigated for ovarian toxicity. Additive or synergistic effects of multiple ovotoxicants present in PM_2.5_ in defined mixtures have been investigated to a very limited extent to our knowledge. Two studies by one group have investigated the effects of two PAH mixtures in cultured granulosa cell lines. One mixture (M1) contained all 16 USEPA priority PAH pollutants and the other (M2) contained the 5 most abundant of the 16 at concentrations found in human maternal and cord blood; the PAHs in M2 are lower molecular weight and are not classified as human carcinogens [43, 72]. The authors reported that the effects of the two mixtures on gene expression, cell proliferation, and apoptosis activation differed from one another and differed between a “normal” granulosa cell line and a granulosa cell tumor line; however, the experiments were not designed to differentiate between additive or synergistic effects of the compounds in the mixtures [19, 43].

We observed that the percentage of mice with irregular estrous cycles increased in the PM_2.5_ exposed group. The estrous cycle is regulated by the hypothalamic-pituitary-ovarian axis [73]. After ovulation, if fertilization does not occur, a new cohort of antral follicles begins to grow. These follicles produce estradiol, which exerts negative feedback on the hypothalamus and the pituitary gland to inhibit synthesis and secretion of gonadotropin releasing hormone and the gonadotropins (luteinizing hormone, LH and follicle stimulating hormone, FSH), respectively [73]. As the antral follicles grow, they produce more estradiol. Once estradiol surpasses a threshold, it exerts a positive feedback on the hypothalamus and pituitary gland, triggering the preovulatory surges of LH and FSH on the evening of proestrus of the estrous cycle. We observed increased apoptosis of antral follicles in PM_2.5_ compared to Air-exposed mice in the present study, but this was not associated with significantly decreased ovarian estradiol content in the PM_2.5_-exposed mice.

Only a few epidemiological studies have examined the effects of air quality on female reproduction other than pregnancy outcomes. One study found that conception rates from assisted reproduction decreased with higher modeled exposure to PM_2.5_ during embryo culture in the IVF laboratory [74]. Risk of menstrual irregularity during high school increased with increasing exposure to total suspended particulate matter during high school in women in the Nurses Health Study II [75]. Prior to our study, very little experimental research had been conducted on the effects of exposure to particulate matter air pollution on ovarian follicles [76]. In a recent study, Zhou et al. exposed female C57BL/6 J mice via inhalation to ambient PM_2.5_ (median concentration of 36.3 μg/m^3^) or filtered air for 4 months, 12 h/day [30]. Consistent with our results in *Apoe-/-* mice, they reported statistically significant increases in the percentage of mice with irregular estrous cycles and decreases in primordial follicle numbers. In contrast to our results, they also reported statistically significantly decreased antral follicle numbers, and they did not report statistically significant decreases in primary or secondary follicle numbers. These differing results may be due to different follicle counting methodology used and that they did not euthanize the mice on the same estrous cycle stage. It is well-established that antral follicle and corpora lutea numbers vary with estrous cycle stage [53, 77]. Two experimental studies by one group exposed wild type Kunming [29] or ICR [78] mice to PM_2.5_ suspended in saline solution by tracheal instillation and reported increased oxidative DNA damage, increased levels of the proapoptotic BAX protein, and caspase 3 activation in the ovaries. However, these studies had several limitations—the tracheal instillation route is not relevant to humans, the total and daily doses were very high, the follicle and cell types positive for the DNA damage and apoptosis markers were not identified, and no follicle staging or enumeration was performed. Several studies have examined the effects of prenatal exposure to diesel exhaust on ovarian follicles. Exposure to diesel exhaust for one hour daily at target concentration of 600 μg/m^3^ during pregnancy and/or postnatally for the first two months of life was reported to decrease the fractional area of the ovary taken up by primordial follicles in mice [28]. The fractional area taken up by primary follicles was reportedly decreased in the prenatal only exposure group compared to the other three groups, while secondary and antral follicle area did not differ among groups [28]. We note that this study was limited in that follicle area was only assessed in 10 nonoverlapping fields at 100 × magnification per ovary, and how these fields were chosen was not described [28]. Another study examined the effects of prenatal exposure of rabbits to diesel exhaust containing 1 mg/m^3^ of PM_2.5_ for 2 h/day, 5 days/week from days 3 to 28 of gestation on ovarian follicles in the offspring at 7.5 months of age [79]. Follicles were counted in five sections per ovary, and results were expressed per unit area. No statistically significant effects on follicle density were observed [79]. These studies were limited in that neither utilized nonbiased stereological methods to assess ovarian follicle numbers [80, 81].

*Apoe-/-* null mice used in this study lack the APOE cholesterol transport protein, which mediates binding of low-density lipoproteins (LDL) and very low-density lipoproteins (VLDL) to LDL receptors in liver and other tissues, including the ovaries. *Apoe* is strongly expressed in ovarian theca cells, interstitial cells, and corpora lutea of rats, mice, and primates [10–14]. APOE regulates androgen synthesis in cultured rat theca cells via regulation of expression of P450 17α-hydroxylase, C17-20 lyase (*Cyp17a1*), the rate-limiting enzyme for androgen synthesis, which converts progesterone to androstenedione [15]. We included control ovaries from wild type mice known to be in proestrus (cycle stage with highest estradiol levels) with the experimental ovaries in our estradiol assays. Interestingly, we noted that these wild type mice all had high ovarian estradiol content, while only some of the *Apoe-/-* Air or PM_2.5_-exposed mice had comparable estradiol content, despite also being euthanized on proestrus. This suggests that *Apoe-/-* mice have a deficit in estradiol synthesis, which is consistent with one other publication that reported lower serum estradiol concentrations in *Apoe-/-* mice on the day of diestrus (cycle stage with low estradiol levels) at 100 days of age compared to non-littermate C57BL/6 J mice [10].

Very little is known about the roles of APOE in primordial and primary follicles. One study reported that primordial follicle numbers were increased in *Apoe-/-* mice compared to wild type mice prior to puberty, while there were significantly fewer primordial follicles at 100 days of age [10]. However, non-littermate wild type C57BL/6 J controls from a different supplier than the *Apoe-/-* mice were used in that study. The same study reported that fertility of *ApoE-/-* females is not significantly different from the wild type C57BL/6 J controls with respect to total numbers of pups, number of litters or average number of pups per litter, over a reported 6-month breeding study [10]. We cannot exclude that *Apoe* deletion may have increased ovarian sensitivity to PM_2.5_ exposure in the present study. Therefore, in future studies, we plan to compare the ovarian effects of *Apoe* deletion and exposure to PM_2.5_ alone and in combination.

*Apoe-/-* null mice develop hyperlipidemia and atherosclerosis, with fatty streaks developing at 3 months of age, atherosclerotic plaques evident by 20 weeks of age, and progression of lesions over time [22, 23]. Prior studies have reported that exposure to PM_2.5_ exacerbates atherosclerotic plaque development in male *Apoe-/-* mice [24, 25, 82], but to our knowledge no published studies have investigated the effects of PM_2.5_ on this endpoint in female *Apoe-/-* mice. We hypothesized that PM_2.5_-induced decline in ovarian function would exacerbate atherosclerotic effects of PM_2.5_ in females and therefore that ovariectomized females, which lack all ovarian function, would experience more pronounced cardiovascular effects of PM_2.5_ than ovary-intact females. Consistent with our hypothesis, there were statistically significant effects of ovariectomy and PM_2.5_ exposure on two measures of atherosclerosis progression, lumen area and percentage of endothelial cell area positive for the lipid marker Oil Red O. The percent lumen area was smaller in the PM_2.5_ exposed SHAM mice than all the other groups, and the percentage of positive endothelial cells was increased by OVAX and PM_2.5_ alone. However, the combined effect of OVAX plus PM_2.5_ exposure on Oil Red O positive endothelial cell area was less than additive. The effect of ovariectomy alone is consistent with a prior study, which reported larger and more extensive atherosclerotic plaques in ovariectomized female *Apoe-/-* mice compared to ovary-intact controls [22]. A possible mechanism is suggested by a report that estradiol increases nitric oxide and protein *S*-nitrosylation in cultured primary vascular smooth muscle cells; the authors postulated this may play a role in the higher levels of plasma nitric oxide and protein *S-*nitrosylation and lower susceptibility to atherosclerosis in female compared to male *Apoe-/*- mice [83]. With regard to heart rate and heart rate variability, both heart rate and RMSSD increased with PM_2.5_ exposure in both OVAX and SHAM females in the 2018 cohort, but the change in RMSSD compared to baseline was statistically significant only in the OVAX mice.

Effects of PM_2.5_ exposure on blood pressure differed depending on whether mice had implanted transmitters and if they had transmitters whether blood pressure was measured via the transmitter or via tail cuff. Transmitter blood pressure measurements were not affected by PM_2.5_ exposure in OVAX mice, but transmitter blood pressure was decreased in SHAM mice. In contrast, tail cuff SBP was increased with PM_2.5_ exposure in OVAX mice without transmitters, while blood pressure was not affected in SHAM mice without transmitters. An effect of transmitter implants on blood pressure was further supported by males with transmitters having lower tail cuff blood pressures and females with transmitters having lower tail cuff blood pressures than mice of the same sex without transmitters during the baseline measurement periods. These differences were no longer statistically significant during the last 6 weeks of exposure, which could suggest that they may have been prolonged effects of the stress of the implantation surgery.

In agreement with a previous study [42], we observed no effect of PM_2.5_ exposure on heart rate in male *Apoe-/-* mice. Female mice were not included in that prior study. Keebaugh et al. [42] also observed decreases in both low frequency heart rate variability and high frequency heart rate variability, indicating decreased parasympathetic influence on the heart. Consistent with those prior findings, PM_2.5_ exposure was associated with a significant decrease in RMSSD, a measure of parasympathetic inputs to the heart, for gonad-intact males and females in the 2017 cohort of the present study. In contrast, we observed statistically non-significant decreases in SDNN and significant increases in heart rate in ovary-intact and ovariectomized females and significant increase in RMSSD in ovariectomized females in the 2018 cohort. Stresses related to the ovariectomy and sham surgeries in the 2018 cohort may have influenced blood pressures and HRV outcomes, making it difficult to compare results from ovary-intact mice between the 2017 and 2018 cohorts. However, we observed no cohort effect on ovarian follicle counts.

We observed that blood pressures measured via tail cuff were generally higher than those measured via telemetry. It has previously been reported that both SBP and DBP increase in male and female mice immediately upon handling or movement of their cage by about 50 and 30 mmHg, respectively, and slowly return to baseline over a period of about one hour [72]. In our study, mice were acclimated for approximately 10 min to the restraining tube used for tail cuff blood pressure measurement prior to measurement onset. Therefore, our results are consistent with those of Wilde et al. [72] in showing increased blood pressure in mice after 10 min of restraint.

## Conclusions

In conclusion, inhalational exposure of adult female *Apoe-/-* mice to concentrated ambient PM_2.5_ at environmentally relevant concentrations depleted ovarian primordial, primary, and secondary follicles by 45%, 40%, and 17%, respectively. The effects of PM_2.5_ exposure on ovarian follicle numbers were consistent between two consecutive exposure years. Our data support that the mechanism of follicle depletion involves increased recruitment of primordial follicles into the growing pool and induction of DNA damage and apoptotic death in growing follicles. Both exposure to PM_2.5_ and ovariectomy increased atherosclerotic plaque progression compared to intact, non-ovariectomized females. However, the effects of both interventions were not additive; plaque increases in PM_2.5_-exposed OVAX mice were slightly higher than in PM_2.5_-exposed SHAM mice but the difference was not statistically significant. Because primordial follicles are non-renewable, their depletion causes premature ovarian failure. In addition to causing infertility, premature ovarian failure, often called premature menopause, in women is associated with increased risk of developing cardiovascular disease, osteoporosis, and Alzheimer’s disease [5–8]. Therefore, our results suggest that exposure to PM_2.5_ could increase women’s risk for these chronic diseases by hastening the onset of menopause.

## Methods

### Chemicals and reagents

All chemicals and reagents were obtained from Sigma Aldrich (St. Louis, MO) or Fisher Scientific unless otherwise noted.

### Animals

The experiments were conducted using genetically modified mice in which part of exon 3 and part of intron 3 of the *Apoe* gene were deleted, resulting in no expression of APOE protein (B6;129P2-*Apoe*^*tm1Unc*^/J; hereafter referred to as *ApoE-/-*) [84]. The mice were bred in house by mating *ApoE-/-* females and males from stock originally purchased from Jackson Labs, where they were backcrossed at least 10 generations onto a C57BL/6 J genetic background (Strain # 002052; https://www.jax.org/strain/002052). This mouse strain was chosen because it is a model of choice for the investigation of atherosclerosis [23] because wild type mice do not develop atherosclerosis. Mice were housed in an Association for Assessment and Accreditation of Laboratory Animal Care International (AAALACI)-accredited vivarium on a 12-h light/12-h dark cycle with food and water available ad libitum. Temperature was maintained at 70 °C and relative humidity averaged 48%. The experiments described herein were performed in three cohorts, one in 2017, one in 2018, and one in 2019. In 2017 gonad-intact male and female mice were studied; in 2018 ovary-intact SHAM and ovariectomized (OVAX) female mice were studied to investigate the effects of ovariectomy on the cardiovascular parameters; in 2019 ovary-intact females were studied to examine the effects of PM_2.5_ exposure on ovarian estradiol concentrations. PM_2.5_ concentrations were slightly different from year to year but the mean concentrations were not significantly different (Table 1). The experimental groups, ages at surgeries and exposure onset, and endpoints analyzed for each year are shown in Table 9.

**Table 9:** Experimental design

	2017 cohort	2018 cohort	2019 cohort
Exposure months	March–June	August–November	August–November
Age at first surgery	3 months	3 months	3 months
Age at start of exposure	4 months	4 months	4 months
Exposure duration	12 weeks	12 weeks	12 weeks
Experimental groups	Gonad intact male Air Gonad intact male PM_2.5_ Gonad intact female Air Gonad intact female PM_2.5_	Sham female Air Sham female PM_2.5_ OVAX female Air OVAX female PM_2.5_	Sham female Air Sham female PM_2.5_
Endpoints	Male and female	Sham and OVAX	Sham
	BP (tail cuff and transmitter)	BP (tail cuff and transmitter)	Ovarian estradiol Estrous cycling
	ECG (transmitter)	ECG (transmitter)	
		Oil red O aorta	
	Female only	Sham only	
	Estrous cycling	Ovarian estradiol	
	Follicle counts	Estrous cycling	
	Ovarian IHC (PUMA, activated caspase 3, γH2AX, Ki67)		
	Ovarian TUNEL		

#### Surgery and biopotential data collection

Ovariectomies were performed when the mice were 12 weeks of age via small bilateral incisions about 5 mm below the rib cage under isoflurane anesthesia [85]. Sham ovariectomized mice underwent an identical procedure, except ovaries were not excised. Mice were provided subcutaneous ketoprofen for 2 days after surgery for analgesia and subcutaneous enrofloxacin for 4 days.

At 3 months of age in the 2017 cohort, and 2 weeks after ovariectomy or sham surgery in the 2018 cohort, 4 or 5 mice from each experimental group were implanted with telemetry devices (HD-X11, Data Sciences International) which allow collection of electrocardiograms (ECG), blood pressure (BP), body temperature and activity. ECG leads were placed in a lead II configuration. Blood pressure was measured through a pressure transduction catheter inserted into the right carotid artery. Mice were provided subcutaneous analgesia after surgery, buprenorphine for 2 days in the 2017 study or ketoprofen for 3 days in the 2018 study, and subcutaneous enrofloxacin for 7 days. After surgery, we allowed for two weeks of recovery followed by one week of acclimation to the exposure chambers and one week of baseline exposure to filtered air. Data from the telemetry devices were recorded, using the iox2® (EMKA Technologies, Falls Church, VA, USA) acquisition system, starting 6 h after the exposure period, overnight when undisturbed in housing.

#### Heart rate variability and blood pressure

Inter-beat intervals (RR) were extracted from ECG recordings and analyzed for heart rate variability (HRV) measures using ecgAUTO® (EMKA Technologies, Falls Church, VA, USA). HRV measures in the time-domain, standard deviation of normal RR intervals (SDNN) and root mean square of successive differences of normal RR intervals (RMSSD), indicate the magnitude of variance in the heart’s rhythm. SDNN represents total HRV, while RMSSD is conventionally interpreted as an indicator of the heart’s response to parasympathetic, or vagal, inputs [86]. HRV was assessed in 3-min epochs at 30-min intervals [87] from 19:00 to 24:00 each night. Intra-arterial blood pressure was recorded simultaneously. To normalize for inter-individual variability, HRV and blood pressure data were analyzed in terms of percent changes from baseline for each respective animal and averaged by exposure group and sex or treatment group. Blood pressure data were also analyzed without this transformation. To assess chronic exposure-related changes of HRV and BP from the long-term studies, the final 6 weeks were grouped.

Blood pressure was also measured using the CODA HT-8 tail cuff blood pressure system system (Kent Scientific) once per week, except during the final week of exposure, in mice with and without implanted transmitters in 2017 and in mice without transmitters only in 2018. Mice were acclimated in the restraint tube for 10 min prior to blood pressure measurement.

### Exposure to PM_2.5_

Starting at 4 months of age, mice were exposed to concentrated ambient PM_2.5_ for a cumulative total of 240 h, nominally 5 h per day, 4 days per week for 12 weeks adjusted as needed for technical issues and holidays. Exposures were performed using a Versatile Aerosol Concentration Enrichment System (VACES) [88–90] coupled with custom-designed individual exposure 12.4 cm high × 77.2 cm^2^ compartments within a larger chamber [91] on the University of California Irvine campus near the intersection of two heavily trafficked roadways. Control mice were exposed to filtered air (Air) under conditions identical to the animals that were exposed to PM_2.5_. The exposures reported herein were conducted March through June 2017, August through November 2018, and August through November 2019.

All mice were euthanized at 7 months of age, 24 h after the last exposure. Ovary-intact female mice were euthanized on the day of proestrus of the estrous cycle, based on vaginal cytology containing a mixture of nucleated and cornified epithelial cells, 24 h after the last exposure. Mice were first anesthetized with isoflurane, then underwent exsanguination by cardiac puncture. For both the 2017 and 2018 cohorts, one ovary with attached oviduct was fixed in Bouin’s fixative overnight at 4 °C for stereology. For the 2017 cohort the other ovary with attached oviduct was fixed in 4% paraformaldehyde in phosphate buffered saline (PBS) overnight at 4 °C followed by cryoprotection in 30% sucrose in PBS until the tissue sank; these ovaries were used for immunostaining. For the 2018 cohort the second ovary and for the 2019 cohort both ovaries were snap frozen and stored at -80 °C for subsequent estradiol measurements. For the 2018 and 2019 cohorts, aortas were dissected and fixed in 4% paraformaldehyde for analysis of atherosclerosis.

Male mice and ovariectomized females were euthanized in the same manner 24 h after the last exposure.

### Size-resolved particle number and mass concentrations

A TSI Scanning Mobility Particle Sizer (SMPS, Shoreview, MN, USA) was used to measure the ultrafine particle fraction of PM_2.5_ and particles up to about 1 μm. An optical particle counter was used to measure particle concentrations in the size range of 0.5 μm to 2.5 μm. In addition to monitoring particle mass, a TSI condensation particle counter (Model 3022) was run in parallel to measure total particle number concentrations. A TSI DustTrak optical mass monitor (Model 8520) was used to provide integrated PM_2.5_ mass concentrations.

### Estrous cycle monitoring

For evaluation of estrous cycling, female mice were individually housed, and vaginal lavage using 0.9% NaCl [92] was performed every morning for at least 14 days. Cells in the lavage fluid were examined by light microscopy immediately after collection, and the predominant cell types present in the fluid were recorded. Mice with telemetry implants were excluded from analyses of the effect of PM_2.5_ exposure on estrous cycling and ovarian endpoints to exclude any effects of the implants on these endpoints.

### Ovarian follicle counts

Bouin’s fixed ovaries from mice without telemetry implants were embedded in glycolmethacrylate resin (Technovit 8100; Heraeus Kulzer GmBH, Wehrheim, Germany), sectioned at 20 µM, and stained with hematoxylin and eosin.

Stereological methods were used to obtain unbiased estimates of ovarian follicle numbers [81, 93]. Ovarian follicles were counted blind to the treatment group using Stereo Investigator software (MBF Bioscience) with an Olympus BX40 light microscope equipped with 4 × UPlanFl, 10 × Plan, 40 × PlanApo N340 and 60 × PlanApo objectives, a joystick controller for a motorized XY stage (Ludl Electronic Products), and an Optronics MicroFire digital camera. We used the fractionator/optical dissector method to obtain unbiased and efficient estimates of primordial and primary follicle numbers by counting follicles in a defined fraction of the whole ovary [81]. Three levels of sampling were used to determine the estimated number of primordial and primary follicles in the ovary. For both the 2017 and 2018 ovary cohorts, follicles were counted in 6% of the ovarian volume. The first level of sampling was at the level of the sections – in 2017 every 3^rd^ section was counted and in 2018 every 5th section of the ovary was counted. The second level of sampling was a fraction of the area of each section. In 2017, 45 μm × 45 μm counting frames were superimposed onto the sections in sampling grids that were subdivided into 90 μm × 90 μm squares, while in 2018 the counting frames were 175 μm × 175 μm within 250 μm × 250 μm squares. Follicles were counted if the oocyte fell within the counting frame and/or touched the inclusion boundaries and did not touch the exclusion boundaries. Lastly, the optical dissector height was set to 8 out of 13 μm (2017) or 10 out of 17 μm (2018) with guard zones on the top and the bottom of the section to account for irregularities of the sections. By multiplying the raw counts by the reciprocal of the counting fraction, the number of follicles in the entire ovary was estimated. Secondary, antral, and preovulatory follicles, and corpora lutea were followed through every section to avoid counting any of these large structures more than once. The total number of healthy or atretic secondary and antral follicles or corpora lutea was calculated as the sum of the counts. Healthy follicles were classified as primordial (single layer of fusiform granulosa cells), primary (single layer with two or more cuboidal granulosa cells), secondary (greater than one layer of granulosa cells with no antrum), or antral [94, 95]. Atretic follicles were identified as previously described [53, 96, 97].

### Estradiol measurement using LC–MS/MS

We adapted the LC–MS/MS estradiol assay method published by Keksi-Rahkonen et al. [98], which incorporates derivatization of estradiol with 1,2-dimethylimidazole-5-sulfonyl chloride (DMIS).

#### Sample preparation

One ovary per animal from the 2018 cohort and both ovaries from each animal from the 2019 cohort were suspended in ice cold 325 µL of 0.5% bovine serum albumin (BSA) + 5 mM EDTA pH7.4. Bilateral ovaries from proestrous, wild type C57BL/6 J mice were included as assay positive controls, as this is the cycle stage when ovarian estradiol concentrations are highest [99]. Ovaries were homogenized on ice using a Kontes mortar and pestle apparatus for 30 s per ovary, or until ovarian tissue was fully dissociated. Samples were then centrifuged at 15,800 × g for 10 min and 300 µL of supernatant was aliquoted into separate microfuge tubes. Blanks were prepared using 300 µL of 0.5% BSA + 5 mM EDTA. An aliquot of the internal standard, d4-17β-estradiol (5 ng/mL), was spiked into each ovary sample (2.5 µL) and BSA blanks (5 µL). Calibration curve standards were made using 100 µL of a stock solution of 17β-estradiol diluted to 100 ng/mL in 0.5% BSA + 5 mM EDTA. The blanks, ovary samples, and calibration standard were all suspended in 1 mL of 3:2 hexanes: ethyl acetate then vortexed vigorously for 30 s per sample, inverting periodically. All samples were then centrifuged at 15,800 × g for 5 min and 950 µL of the organic phase was aliquoted into new microfuge tubes. A fresh aliquot of 500 µL of deuterated 17β-estradiol (d4-17β-estradiol, 5 ng/mL) was made to assess percent recovery. All samples, including the internal standard aliquot were evaporated to dryness under N_2_ gas. Then all samples, blanks, and standards were suspended in 50 µL of derivatizing agent, DMIS (1,2-dimethyl-1H-imidazole-4-sulfonyl chloride), at 1 mg/mL in acetone then 100 µL of 50 mM of sodium bicarbonate pH 10.5 was added and incubated at 60 °C for 15 min. Following incubation, samples were resuspended in 343 µL of ethyl acetate and vortexed vigorously, then centrifuged at 15,800 × g for 5 min and 330 µL of the organic phase was aliquoted into fresh tubes. All samples, blanks, and standards were then evaporated to dryness under N_2_ gas and resuspended in 500 µL of methanol for the standards, 150 µL for the blanks, and 75 µL for ovary samples. Finally, the calibration curve standards were prepared by successive dilutions of the stock solution of the derivatized 17β-estradiol and each final standard solution (291 µL) was spiked with 9 µL of the d_4_-DMIS internal standard stock solution in methanol.

#### LC–MS/MS

The extracts (50-µL injection volume) were analyzed using a UPLC-PDA-MS platform (Waters) equipped with an Acquity H Class PLUS system (including a quaternary solvent manager, a sampler manager using a flow-through needle mechanism, a column heater and a photodiode array detector) coupled to a Xevo TQD triple quadrupole mass spectrometer. The separation was achieved using an Acquity UPLC BEH C18 1.7 µm, 50 $\times$ 2.1 mm (Waters) column fitted with a VanGuard pre-column (UPLC BEH C18, 1.7 µm 5 × 2.1 mm; Waters) and maintained at 40 °C. The mobile phase combination was (A) 0.2% acetic acid (99.7%, Optima LC–MS; Fisher) in 18.2 MΩ cm milliQ water (Millipore; model ZD5211584) and (B) LC–MS grade methanol (Optima LC–MS; Fisher). The eluent gradient was as follows: 0–6 min linear gradient from 30% B to 95% B, 6–8 min hold at 95% B, 8–12 min linear gradient back to 30% B and finally 12–14 min hold at 30% B with a flow rate of 0.3 mL min^−1^. UV–vis absorption spectra were acquired using a photodiode array detector (PDA eLambda; Waters) over the full 190–500 nm wavelength range. Exiting the PDA, the samples were introduced into the mass spectrometer using an atmospheric pressure photoionization source (APPI; Waters). Toluene (OmniSolv; EMD) was combined with the eluent post-column at a flow rate of 40 µL min^−1^ and used as an ionization dopant from 5–8 min. APPI source conditions were as follows: repeller voltage, 3.00 kV; cone voltage, 50 V; APPI probe temperature, 500 °C; desolvation gas flow rate, 250 L hr^−1^; source temperature, 150 °C. The analysis was performed in positive ion mode using a multiple reaction monitoring (MRM) approach. The m/z 431 =  > m/z 96 transition was used for the derivatized estradiol product (E2-DMIS) while the m/z 435 =  > m/z 96 transition was used for deuterated derivatized estradiol internal standard (d_4_-DMIS) [98]. The data were acquired using MassLynx software (Waters) and then batch-processed using TargetLynx for quantitative analysis (Waters) using an internal calibration method.

Extraction recoveries were evaluated weekly using the average d_4_-DMIS internal standard concentrations from the spiked ovary samples and comparing them to the d_4_-DMIS concentrations in the spiked calibration standard solutions. From those, recoveries ranging from 80 to 89% were determined and applied to the ovary samples.

### Immunohistochemistry

Ovaries fixed in 4% paraformaldehyde were cryoprotected in 30% sucrose in PBS at 4 °C, embedded in Optimal Cutting Temperature (OCT) compound, and stored at -80 °C before being serially sectioned using a cryostat at a thickness of 10 µm. Sections were mounted 4 per slide in 4 sets so that sections on a slide were separated by 40 µm from one another in the ovary. Immunohistochemistry was performed using the Vectastain ABC kit (PK-4001; Vector Laboratories, Burlingame, CA, USA). Briefly, sections were thawed and heated for 15 min at 95 °C in a 10 mM citrate buffer (pH 6.0). The primary antibodies—rabbit anti-cleaved caspase-3 Asp 175 (1:100; Cell Signaling #9661, Beverly, MA, USA), rabbit anti-Ki67 (1:500; Abcam #15580, Cambridge, MA, USA) and rabbit anti- phosphorylated histone 2AX (γH2AX) (1:200; Cell Signaling #9718)—were detected using biotinylated goat anti-rabbit secondary antibody in 5% normal goat serum. All immunostaining procedures included avidin/biotin and 3% hydrogen peroxide blocking steps. Peroxidase activity was visualized using 3,3´-diaminobenzidine (DAB) as substrate (Roche). Sections were counterstained with hematoxylin. The following negative controls were included in every experiment: secondary antibody without primary antibody; primary antibody without secondary antibody; and primary antibody replaced by rabbit IgG with secondary antibody.

The numbers of follicles with oocytes or granulosa cells positive or negative for Ki67, cleaved caspase-3, and γH2AX immunostaining were counted in 3 or 4 slides (12–16 sections, 4 sections/slide) per endpoint distributed throughout the ovary by an investigator blind to exposure group using an Olympus BX-60 microscope with 10, 20, and 40 × U PLAN FLUO objectives equipped with a Retiga 2000R cooled CCD digital camera system with Q-Capture Pro software. The fractions of positive primordial and primary follicles (containing one or more positive granulosa cells per largest cross-section or containing positive oocytes) and secondary and antral follicles (containing three or more positive granulosa cells per largest cross-section or containing positive oocytes) were calculated and used for data presentation and analysis.

### Terminal deoxynucleotidyl transferase dUTP nick end-labeling (TUNEL)

TUNEL was carried out using the In Situ Cell Death Detection Kit POD (Roche) as previously described [94]. Negative controls without terminal transferase and positive controls treated with DNAse I were included in every experiment. The numbers of TUNEL positive and negative secondary and antral follicles (containing 3 or more TUNEL positive granulosa cells per largest cross-section) were counted in 12 sections (3 individual runs, 4 sections/run) distributed throughout the ovary blind to experimental group. Primordial follicles never and primary follicles almost never had TUNEL positive cells. The percentages of TUNEL positive secondary and antral follicles were calculated and used for data presentation and analyses.

### Arterial histology

The aortic root was embedded in OCT, stored frozen (-80◦C), cryosectioned at 10 µm, stained with Oil Red-O and counterstained with hematoxylin to measure the degree of plaque formation. Images of the aortic root were acquired at 10 × magnification, recorded and digitized. ImageJ software (NIH) was used to outline both the total endothelial layer area and the Oil Red O-positive endothelial cell area for each section and expressed as percent Oil Red-O area. For each slide, the total cross-sectional area and the unobstructed lumen area were measured using ImageJ; the percent lumen area was determined. Slides from 3 to 4 individual mice in each exposure group were averaged across all the sections for each animal for data analysis and presentation.

### Statistical analyses

Data are presented as means ± SEM unless otherwise noted. Follicle count and estrous cycle data were analyzed by 2-way ANOVA with exposure and year of experiment as independent variables. Immunostaining data expressed as fractions were analyzed by non-parametric Mann Whitney U test or fractions were arcsine square root transformed and analyzed by t-test [100]. The results of the two different analyses were very similar and are presented for all endpoints. Heart rate variability and blood pressure variables were analyzed using independent samples t-test following assessment of equality of variances using Levene’s test with significance assessed at *P* ≤ 0.05. Statistical analyses were performed using SPSS 25 for MacIntosh or SPSS for Windows (IBM Corporation, Armonk, NY, USA).

## Supplementary Information

**Additional file 1.** Effects of PM2.5 exposure on changes in systolic and diastolic blood pressure from baseline.

## Data Availability

The datasets used during the current study are available from the corresponding author on reasonable request.
