# Supplementary material for: Exposure to environmentally relevant concentrations of ambient fine particulate matter (PM2.5) depletes the ovarian follicle reserve and causes sex-dependent cardiovascular changes in apolipoprotein E null mice
Source: Part Fibre Toxicol. 2022 Jan 7;19:5. doi: 10.1186/s12989-021-00445-8 (PMC8740366; doi:10.1186/s12989-021-00445-8)
Supplement: Supplementary file 1 — Additional file 1. Effects of PM2.5 exposure on changes in systolic and diastolic blood pressure from baseline. [file 12989_2021_445_MOESM1_ESM.docx]

**SUPPLEMENTAL DATA**

**Effects of PM_2.5_ Exposure on Change in Blood Pressure from Baseline**

**Methods:** A subset of 4-5 animals per group underwent surgery for subcutaneous implantation of a radiotelemetry device with lead-II electrocardiogram (ECG) leads and a pressure transduction catheter inserted into the right carotid artery allowing for internal measurement of blood pressure (BP). ECG and BP data was collected overnight in housing throughout the exposure period.

All statistical analyses performed were Student’s t-test using all data points for each animal’s daily BP measures throughout the specified averaging time.

**Results: Effects of PM_2.5_ Exposure on Change in Blood Pressure from Baseline for 2017 cohort, Male v Female**

Results: Blood pressure was analyzed during the final 5 weeks of the exposure, weeks 8-12, as a change from baseline (Table 1). Male animals exposed to concentrated ambient PM_2.5_ showed a significant drop in diastolic BP as compared to air-exposed, however there was no difference in systolic BP. PM_2.5_ exposure in female ApoE-/- mice led to an increase in both systolic and diastolic blood pressures, which is statistically different than the decrease seen in air-exposed females.

***Table 1: Effects of exposure to*** *P****M_2.5_ on change from baseline intra-arterial blood pressure in gonad-intact males and females***

|  |  | Male | | |  | Female | | |
| --- | --- | --- | --- | --- | --- | --- | --- | --- |
|  |  | Air | PM_2.5_ | p-value |  | Air | PM_2.5_ | p-value |
| Systolic | *mmHg* | -3.7 ± 1.5 | -2.8 ± 1.1 | 0.616 |  | -13.1 ± 1.8 | 6.1 ± 0.9 | <0.001 |
| Diastolic | *mmHg* | -1.7 ± 0.9 | -5.5 ± 1.0 | 0.009 |  | -13.6 ± 1.8 | 4.3 ± 0.9 | <0.001 |

Table 2. *Change from baseline of intra-arterial blood pressure, averaged during the final 5 weeks of exposure (weeks 8-12) of ovariectomized and sham female mice exposed to filtered air or* PM_2.5_

**Results: Effects of PM_2.5_ Exposure on Change in Blood Pressure from Baseline for 2018 cohort, Ovariectomized v Sham**

Blood pressure was analyzed during the final 5 weeks of exposure, weeks 8-12, as a change from baseline to depict the effect of chronic PM_2.5_ exposure on ovariectomized female ApoE-/- mice and sham ApoE-/- controls (Table 2). In ovariectomized mice, PM_2.5_ exposure did not significantly change either systolic or diastolic or diastolic blood pressure as compared to air exposure. There was a significant difference in both systolic and diastolic blood pressure between PM_2.5_- and air-exposed sham animals, where PM_2.5_ led to a decrease in BP, and air exposure showed an increase in BP over time.

***Table 2: Effect of exposure to*** *P****M_2.5_ on change from baseline intra-arterial blood pressure in ovariectomized or sham-operated females***

|  |  | Ovariectomized | | |  | Sham | | |
| --- | --- | --- | --- | --- | --- | --- | --- | --- |
|  |  | Air | PM_2.5_ | p-value |  | Air | PM_2.5_ | p-value |
| Systolic | *mmHg* | 3.4 ± 0.7 | 3.0 ± 0.6 | 0.690 |  | 9.1 ± 0.6 | -4.4 ± 2.0 | <0.001 |
| Diastolic | *mmHg* | 6.6 ± 0.8 | 5.4 ± 0.7 | 0.215 |  | 3.2 ± 0.6 | -1.1 ± 1.9 | 0.008 |

Table 2. *Change from baseline of intra-arterial blood pressure, averaged during the final 5 weeks of exposure (weeks 8-12) of ovariectomized and sham female mice exposed to filtered air or* PM_2.5_

Changes from baseline blood pressure differed significantly between ovariectomized and sham mice within the exposure groups (Table 3). In air-exposed groups, systolic BP increased less in ovariectomized compared to sham animals, whereas diastolic BP increased significantly more in ovariectomized animals compared to sham mice. In PM_2.5_-exposed animals, however, both systolic and diastolic blood pressure increased over the course of the 12-week exposure in ovariectomized mice, while both systolic and diastolic blood pressure decreased in sham mice.

***Table 3: Effect of ovariectomy on change from baseline intra-arterial blood pressure in females exposed to Air or PM_2.5_***

|  |  | Air | | |  | PM_2.5_ | | |
| --- | --- | --- | --- | --- | --- | --- | --- | --- |
|  |  | Ovax | Sham | p-value |  | Ovax | Sham | p-value |
| Systolic | *mmHg* | 3.4 ± 0.7 | 9.1 ± 0.6 | <0.001 |  | 3.0 ± 0.6 | -4.4 ± 2.0 | <0.001 |
| Diastolic | *mmHg* | 6.6 ± 0.8 | 3.2 ± 0.6 | <0.001 |  | 5.4 ± 0.7 | -1.1 ± 1.9 | <0.001 |

Table3. *Change from baseline of intra-arterial blood pressure, averaged during the final 5 weeks of exposure (weeks 8-12) of ovariectomized (ovax) and sham female mice exposed to filtered air or* PM_2.5_
